# Measuring Self-Reported Access to Assistive Technology Using the WHO Rapid Assistive Technology Assessment (rATA) Questionnaire: Protocol for a Multi-Country Study

**DOI:** 10.3390/ijerph182413336

**Published:** 2021-12-17

**Authors:** Wei Zhang, Arne H. Eide, Wesley Pryor, Chapal Khasnabis, Johan Borg

**Affiliations:** 1 Assistive Technology Access Team, Health Product Policy and Standards Department, World Health Organization, 1211 Geneva, Switzerland; khasnabisc@who.int; 2 Department of Health Research, SINTEF Digital, N-0373 Oslo, Norway; arne.h.eide@sintef.no; 3 Nossal Institute for Global Health, Melbourne School of Population and Global Health, The University of Melbourne, 3010 Melbourne, Australia; wesley.pryor@unimelb.edu.au; 4 School of Health and Welfare, Dalarna University, SE-791 88 Falun, Sweden; jog@du.se

**Keywords:** access to health services, assistive technology, barriers to access, prevalence of need, prevalence of use, representative population survey

## Abstract

In 2018, the World Health Assembly adopted a resolution on improving access to assistive technology (AT), and mandated the WHO to prepare a global report on assistive technology based on the best available evidence and international experience. As limited data on access to AT at country and global levels were available, there was a need to conduct representative population surveys in order to inform the development of the global report, national AT programs, and global initiatives. The objective of this protocol is to describe a multi-country study of access to assistive technology in six self-reported areas: use, source, payer, satisfaction, unmet need, and barriers. In collaboration with WHO Regional and Country offices, Member States, and other stakeholders, the Assistive Technology Access team in WHO coordinates the study. Data are collected through household surveys using the rapid Assistive Technology Assessment (rATA) questionnaire. Findings from the surveys will be published in the global report.

## 1. Background and Objective

Today, an estimated one billion people need assistive technology (AT) to lead productive, inclusive, and dignified lives, yet only 1 in 10 people globally are believed to have access to the AT they need. Access to AT is essential for many people to maintain and improve function, health, and wellbeing; and to participate in education, work, and social activities. Among people who commonly need AT are older people, people with disabilities, and people living with health conditions or with acute health conditions. As the world’s population lives longer, and the prevalence of noncommunicable diseases increases both in real terms and as a proportion of total health burden, the need for AT will increase and is expected to reach two billion by 2030 [1].

In 2018, a resolution on improving access to assistive technology was adopted at the 71st World Health Assembly (WHA71.8). It requests WHO to prepare a Global Report on Assistive Technology (GReAT) by 2021 [2]. The report should be developed based on the best available evidence, and make recommendations to the Member States in developing national AT policies and programs. The report will also discuss AT in Universal Health Coverage (UHC), and contribute to realizing the aspirations of the UN Convention on the Rights for People with Disabilities (CRPD) [3]. As signatories of the CRPD, countries are to report their activities and results with regards to articles 4 (general obligations), 20 (personal mobility), 26 (habilitation and rehabilitation), and 32 (international cooperation), which explicitly reference AT.

Despite the urgency and the global imperative on improving access to AT, little country-level population data have been collected systematically to demonstrate the access to AT [4,5]. GReAT is an opportunity for a coordinated global effort to provide new policy-relevant information for AT.

In 2018, the WHO Assistive Technology Access (ATA) team proposed the first draft rapid Assistive Technology Assessment (rATA) questionnaire to collect data on self-reported access to AT [6]. Self-reporting recognizes the principle that choice and consumer participation are crucial in successful AT implementation [7]. It is necessary to take consumer choice and preference into account as users’ understanding of their need, uptake, use of, and benefit from AT are crucial for developing AT services for all in need. Self-reporting is a feasible and valid survey method for similar topics, especially in resource-limited contexts [8]. The rATA questionnaire was developed based on widely used questionnaires related to disability, rehabilitation, and health system studies, and in consultation with global stakeholders in AT. The rATA addresses access to AT according to the people-centered approach proposed by the WHO [9]. The questionnaire covers six self-reported AT areas: use, source, payer, satisfaction with the products and related services, unmet need, and barriers to access. Source also includes travel distance to source, and satisfaction also includes suitability of assistive products for different environments and activities. Moreover, the questionnaire covers self-reported functional difficulties in six domains: cognition, communication, hearing, mobility, seeing, and self-care [10]. The questionnaire was peer reviewed and tested in two districts in Bangladesh in 2018 [11]. A revision was completed in April 2019, which integrated field testing feedback and research experts’ recommendations as well as further inputs from other relevant technical units within WHO. In May 2019, the Ministry of National Health Services Regulations and Coordination in Pakistan led cognitive and field testing of the revised questionnaire and the full-scale deployment of the survey methodology [12]. In addition, two studies were conducted in Indonesia and Sierra Leone using the same questionnaire to measure access to AT in informal settlements [13]. This tested the usability of the instrument in diverse contexts, providing further evidence of the feasibility of the method, requirements for enumerator training and survey management. In November 2019, a group of AT experts and stakeholders participated in a two-day consultation in Geneva to share and discuss experiences of using the rATA questionnaire. The consultation included experts in research, policy making, program development from both high and low-middle income countries, and WHO regional and country advisors in the relevant subject areas. The participants recommended minor changes to the questionnaire, which was then finalized. The finalized rATA questionnaire was examined for face and construct validity by domain experts on measuring the right constructs in an efficient fit-for-purpose manner. 

In January 2020, WHO launched a global initiative, and called for contributions to data collection on access to AT at the population level, inviting Member States and other AT stakeholders across the world to conduct surveys with the support of WHO. The goals of this multi-country study were to obtain data and evidence on access to AT; to advocate and raise awareness to governments and civil society about the importance of AT; to advance research and development in AT; and to support the design, planning, and prioritization of AT programs or interventions at country and global levels. The study is expected to provide essential data for the development of GReAT, and to understand efforts required to uphold the rights described in the CRPD and other international laws and conventions. The objective of this manuscript is to describe this multi-country study of access to AT.

## 2. Methods

### 2.1. Study Design

Using the rATA questionnaire, the multi-country study will collect self-reported data in representative population surveys in multiple countries across the world. Wherever possible, national household surveys representing the general population are to be conducted. Surveys representing the general population in one or more specific areas in a country will be conducted where national surveys are not feasible due to financial or logistic constraints.

Two-stage cluster sampling is applied in each survey. The first stage is a random selection of geographical units (primary sampling units or enumeration areas), and the second stage is a random selection of households within each chosen geographical unit.

The survey sample size is estimated based on the following formula:

(1) $$n_{h}=\;\frac{\left(z^{2}\right)\left(r\right)\left(1-r\right)\left(f\right)\left(k\right)}{\left(p\right)\left(\tilde{n}\right)\left(e^{2}\right)},$$

where r is the key indicator for the survey, which is the prevalence of access to any assistive product in the target population. Based on the available WHO estimate—one billion people need AT, and only 10% of those in need have access to it—the prevalence of access is estimated around 1% of the population globally. To determine this indicator with 95% (e = 1.96) confidence between 0.75% and 1.25% (e = 25% × r) in the whole population (p = 1), factoring a non-response rate of 10% (k = 1.1) and the design effect f = 2, the minimum required sample size is 13,392 persons. With an average household size of $\tilde{n}$, the number of households to be surveyed in each country is $n_{h}\;$= 13,392/$\tilde{n}$. The required sample size will be adjusted for each survey depending on available budgets, resources, and other constraints in the specific local context.

### 2.2. Participants

The survey targets the general population of all ages in the country or specific areas within the country. Given the purpose of the survey, all members of sampled households are eligible for inclusion. However, only people who consented, or whose guardian consented, are included.

Adult members of the household will be interviewed directly unless they cannot answer the questions on their own (e.g., participants with cognitive impairments). In case of absence, the household will be revisited up to three times to complete the survey. A proxy within the household will be interviewed when individuals are unable to answer. All children under the age of 15 will be interviewed by proxy, normally by the primary caregiver. Children between 12–15 years of age can be present and participate during the interview with the proxy. Children between 15 and 18 years of age can be interviewed on their own, provided that the parents or primary caregiver consents. However, local customs, laws, and organizational policies concerning child protection, in particular, might affect the survey plan in each context.

### 2.3. Consent

A standard, adaptable pro-forma information sheet and consent form is provided to survey teams to use in the data collection (see Appendix A). The survey team lead is responsible to ensure these templates are completed with survey-specific information (e.g., contact person). All enumerators should read the information about the survey as included in the consent form to potential participants. All the participants will be informed about the survey objectives and process, and their right to withdraw at any time during the interview. Participants will give their consent in writing or verbally, in case writing is not an option, prior to the start of data collection. For participants under 18 or those unable to provide consent, a written or verbal consent from a parent or another main caretaker acting on behalf of the parents can be given on behalf of those participants. Consent must be obtained from each participating household member.

### 2.4. Questionnaire

The rATA questionnaire collects data on:

(1) Self-reported use of and unmet need for assistive products;
(2) Sources of, payers for and barriers to accessing assistive products and related services;
(3) Satisfaction with assistive products and related services;
(4) Self-reported functional difficulties; and
(5) Basic demographic information, such as age and gender.

The questionnaire can be administered by trained interviewers. A manual was developed to provide explanation of the rationale and instruction for administration for the questionnaire [14]. Two three-day train-the-trainer workshops were organized by the WHO in November 2020 and February 2021 for master trainers from the country survey teams.

The questionnaire has been translated into Arabic, Chinese, French, Portuguese, Russian, and Spanish by AT experts who were involved in the development, testing, or consultation of rATA. Translation, back-translation into English, and resolution of discrepancies by multiple experts were applied. The questionnaire will be translated into additional local languages if required in the surveyed population.

### 2.5. Data Collection and Management

A mobile application was developed by the WHO using the ERSI/ArcGIS software to support field data collection. Other data collection tools can also be used as long as the data collection process follows the standard procedure described in the manual and training workshops. The mobile application facilitates efficient, standardized data input. The application will store collected data on the device (e.g., mobile phone or tablet), and upload to the secured backend server located in the WHO headquarters when the mobile device is connected to internet. WHO will support each local survey team to validate the mobile application through standardized test cases prior to the enumerator training.

Enumerator training is to be carried out by the local survey team lead based on the guide provided in the manual and the train-the-trainer workshop. Following the training, enumerators will be guided through field testing to practice the data collection process.

To protect participants’ privacy, the survey will only collect de-identified data. Collected data are anonymized at input, as well as in the server with the coded participants’ ID, which will not allow the identification of the participants’ household location or personal identity. Administrative information that may directly or indirectly identify individuals will be re-coded before the survey commences. For example, village and household number will be coded ‘village—A, ‘village—B’, ‘HH—A’, ‘HH—B’. Only authorized local survey team members (e.g., national principal investigator, or authorized personnel by the national principal investigator) will have access to the codebook. The specific address (street number, mailbox number or similar) is not recorded. To facilitate survey fidelity checks and basic geospatial analysis, a low-resolution GPS coordinate is stored. The coordinate is accurate to a 100 m radius, ensuring specific household location cannot be determined from stored data.

Data cleaning and analysis will be conducted by the WHO in collaboration with local survey teams.

### 2.6. Outcome Measures and Analyses

The primary outcomes of the study include the following indicators for each surveyed country or specific surveyed areas in the country.

(1) *Prevalence of use of AT*, which is the proportion of a population using assistive products.
(2) *Prevalence of need for AT*, which is the sum of the prevalence of met need and the prevalence of unmet need. Prevalence of met need is the proportion of a population using assistive products that do not need new or additional assistive products. Prevalence of unmet need is the proportion of a population that needs new or additional assistive products regardless of whether they are already using assistive products or not.
(3) *Met need as proportion of need*, which is the ratio of the prevalence of met need to the prevalence of need.

Associations between the above indicators and the Human Development Index (HDI) of the surveyed country or specific surveyed areas in the country will be analyzed. HDI is a summary measure of average achievement in human development in three key dimensions: health, education, and standard of living [15].

Secondary analyses will determine:

(4) Rank of assistive products in use, in need and with unmet need.
(5) Rank of barriers to accessing needed assistive products.
(6) Distribution of payers for and source of assistive product (including travel distance to source).
(7) Level of satisfaction with the assistive products (including suitability for different environments and activities) and related services (including assessment, training, maintenance and repair).

In the tertiary analyses, primary indicators will be stratified by age, gender, and living context. Additionally, functional profiles between respondents who self-report a need for AT, and those who report no need for AT will be compared to determine and address potential underestimation of population needs. Finally, further analyses will explore the predictive performance of HDI, and its individual dimensions for the primary indicators, namely, the prevalence of use and need, and the met need as the proportion of need for AT.

### 2.7. Follow-up

According to the study goals, the data collection will support the country in developing or improving programs or interventions of access to AT. Countries participating in the surveys will be prepared to provide a follow-up consultation or services to address unmet need for AT identified through the survey. These will be at the individual level, national level, and global level.

At the individual level, the enumerators will be able to provide information on local services for participants who are identified with any potential need for assistive products or services. The information will be delivered to the participants, for example, in a booklet with service and contact details. In countries, where little or no established local service is available, information about assistive products and their benefits will be provided to the participants.

At the national level, the survey outcomes will be first discussed within the country with local AT stakeholders and relevant ministries to develop actional plans for improving access. Further analysis based on the survey data can be conducted to support developing such action plans according to country’s specific context, priority, and available resources.

When the national surveys are completed, a rATA global data collection workshop will be organized by the WHO where all participating countries, as well as WHO regional and country offices, will be invited to share their experiences and action plans. This workshop aims to encourage knowledge exchange, as well as international and regional collaboration for joint development to address the challenges of access to AT.

## 3. Discussion

There are limitations in the study that need to be acknowledged. First, potential inconsistency in the survey translation and interpretation in multiple countries may limit data comparability. Language and culture differences may lead to various interpretations of the same question by participants from different countries. This limitation can be minimized by ensuring the survey methodology follows similar multi-country surveys used for other purposes. This includes following the standard translation process and field conduct process for enumerators, as detailed in the rATA manual. Adaptation to the methodology due to constraints in the local context, e.g., sampling design, should be kept to the minimum. Second, the standardized survey questions may limit country-specific barriers to be captured. To mitigate this, open-ended questions are optional to some topics where country-specific barriers can be captured. Third, self-reported need for AT may not be comparable to clinically collected data.

## 4. Conclusions

The study is expected to contribute to the global AT development through:

- Supporting governments to respond to the World Health Assembly resolution on improving access to assistive technology.
- Making reliable population-level data on the use of and need for AT available to stakeholders, including ministerial bodies, international development agencies, civil society organizations, and researchers, which will improve advocacy on AT, and support global cooperation on improving access to AT.
- Informing stakeholders and the general public about the need for and importance of AT for both individual and community development.
- Providing stakeholders with baseline data on the need and unmet need for, and current access to, AT in populations, offering them a possibility to compare design, planning, or prioritization of AT programs and interventions. It will also enable governments to monitor development in AT use and need in the population over time as an outcome measure of policy interventions.
- Providing an option for standardized data collection about population access to AT.

## Data Availability

Not applicable.

## Appendix A. Informed Consent Template

I am conducting a survey on the access to assistive technology in our country. The purpose of this survey is to understand how many people in our country use assistive technology or need them but do not have access to them, and what the barriers are for people to get the assistive technology. Assistive technology includes commonly known products, such as wheelchairs, glasses, hearing aids, as well as smart phone apps, such as a digital calendar, that support people with difficulties in cognition.

I am going to give you information, and invite you to be part of this survey. Before you decide to participate, you can talk to anyone you feel comfortable with about the survey. The information I am going to give you may contain words that you do not understand. Please ask me to stop as we go through the information, and I will take time to explain.

We will ask everyone who usually lives in your household to do the survey. This survey will involve your participation in an interview to fill out a questionnaire that will take about 15 min per person, on average.

There are about [*#*] participants randomly selected from our country to participate in this survey to represent the access to assistive technology in our population. And you are one of the selected participants from the [*village/city*].

You do not have to participate if you do not want to. If you choose not to participate, it will not affect your access to any future services.

There is a risk that you may share some personal or confidential information by chance, or that you may feel uncomfortable talking about some of the topics. However, we do not wish for this to happen. You do not have to answer any question or take part in the survey if you feel the question(s) are too personal, or if talking about them makes you uncomfortable.

There will be no immediate direct benefit to you, but your participation is likely to help us find out more about how to we can improve the access to assistive technology for yourself, your family, and your community in the future.

The survey being done in the community may draw attention, and if you participate, you may be asked questions by other people in the community. We will not be sharing information about you to anyone outside of the survey team. The information that we collect from this survey will be kept private. Any information about you will have a number on it instead of your name. Only the survey team will know what your number is, and we will lock that information up with a lock and key. It will not be shared with or given to anyone [*optional: adds specific name and organization that will access personal information of the participants*]. You can contact [*name and telephone number/email address*] if you wish to know more about the survey study or have any question any time.

Do you have any questions? Do you feel comfortable to start the survey now?

Certificate of Consent

I have read the foregoing information, or it has been read to me. I have had the opportunity to ask questions about it, and any questions I have asked have been answered to my satisfaction. I consent voluntarily to be a participant in this study.

Print Name of Participant__________________

Signature of Participant ___________________

Date ___________________________

Day/month/year

*If illiterate* (A literate witness must sign (if possible, this person should be selected by the participant, and should have no connection to the research team). Participants who are illiterate should include their thumb print as well.)

I have witnessed the accurate reading of the consent form to the potential participant, and the individual has had the opportunity to ask questions. I confirm that the individual has given consent freely.

Statement by the researcher/person taking consent

I have accurately read out the information sheet to the potential participant, and to the best of my ability made sure that the participant understands that the following will be done:

1. Answering the questions written in the questionnaires in a 10–30 min interview;
2. The participant can withdraw from the interview at any time the participant wishes.

I confirm that the participant was given an opportunity to ask questions about the study, and all the questions asked by the participant have been answered correctly and to the best of my ability. I confirm that the individual has not been coerced into giving consent, and the consent has been given freely and voluntarily.

A copy of this form has been provided to the participant. Print name of researcher/person taking the consent________________________

Signature of researcher/person taking the consent__________________________

Date ___________________________

Day/month/year
